# Morphological and Molecular Variation Between *Fusarium avenaceum*, *Fusarium arthrosporioides* and *Fusarium anguioides* Strains

**DOI:** 10.3390/pathogens7040094

**Published:** 2018-11-29

**Authors:** Tapani Yli-Mattila, Taha Hussien, Olga Gavrilova, Tatiana Gagkaeva

**Affiliations:** 1 Department of Biochemistry, University of Turku, Turku 20014, Finland; taha_bio@yahoo.com; 2 National Research Center, Cairo 80301, Egypt; 3 All-Russian Institute of Plant Protection (VIZR), St. Petersburg-Pushkin 196608, Russia; olgavrilova1@yandex.ru (O.G.); t.gagkaeva@yahoo.com (T.G.)

**Keywords:** beta-tubulin, *F. anguioides*, *F. arthrosporioides*, *F. avenaceum*, ISSR, morphology, pathogenicity

## Abstract

*Fusarium avenaceum* and closely related species are common fungi on various plants, cultivated in different climatic regions. The aim of this study was to determine the taxonomic affiliations of the *F. avenaceum*, *Fusarium arthrosporioides,* and *Fusarium anguioides* strains by using morphological, physiological and molecular-genetic approaches. Twenty-six single-spored morphologically identified strains, which were mainly from cereals, were investigated in order to find out, if they belong to a separate species. Pathogenicity of strains to wheat seedlings and ISSR (Inter Simple Sequence Repeats) fingerprint and beta-tubulin DNA sequence patterns were analyzed. According to phylogenetic analyses, the strains could be divided into two big groups consisting of mostly *F. avenaceum* or *F. anguioides* strains. *F. arthrosporioides* was not detected as a separate species by the sum of the characters. *F. anguioides* was characterized as a separate species, which could be identified by morphological and molecular data. High genetic diversity of the *F. avenaceum* and related species was revealed. One *F. anguioides* strain (rudbeckia, Vladivostok, Russia), had an identical beta-tubulin sequence with two previously sequenced strains of *Fusarium tricinctum* species complex, which were isolated from dicotyledonous plants in Asia.

## 1. Introduction

*Fusarium avenaceum* (Fr.) Sacc., which was described in 1886, is widely distributed and one of the most common *Fusarium* species in plants, which causes damping off, rots of root, stalk and fruit [1,2,3]. It is the causal agent of diseases of cereal crops everywhere, but especially often in temperate climates [4,5,6,7,8]. *F. avenaceum* constitutes an economical problem for growers and is a safety issue due to mycotoxin production in grains, such as moniliformin and enniatins [5,9,10]. The teleomorph of *F. avenaceum* has been described by Cook [11] and Booth and Spooner [12], but the sexual stage of this species has not been found, despite the presence of mating types MAT-1 and MAT-2 being detected [13]. 

*Fusarium anguioides* Sherb. and *Fusarium arthrosporioides* Sherb. are common species on different plants, including cereals and dicotyledons. Both species are often identified as *F. avenaceum* based on the similarity of their morphological data. According to Booth [14] *F. arthrosporioides* has a widespread occurrence, but according to Parikka et al. [15], *F. arthrosporioides* is included in *F. avenaceum* in most scientific papers. *F. anguioides* was not even mentioned in the papers of Nelson et al. [16] and Leslie and Summerell [17], while in the Atlas of Gerlach and Nirenberg [18], *F*. *anguioides* was a member of section Arthrosporiella, characterized by blastic or phialidic conidiogenous cells, very rarely with two conidiogenous loci and forming chlamydospore-like cells. No teleomorph has been described for these two species.

*F. anguioides* and *F. arthrosporioides* were first described by Sherbakoff [19], who placed these two species into the taxonomically assigned section Arthrosporiella Sherb. together with *Fusarium sporotrichioides* Sherb. In Sherbakoff’s opinion, the species of section Arthrosporiella typically form microconidia, which are usually spindle-shaped and 0-3-septate. Sporodochial macroconidia, when present, are sickle-shaped, mostly 5-septate, of section Roseum type, and slightly curved to straight and anguiform; true chlamydospores are absent. According to Sherbakoff [19] the Arthrosporiella section is a connecting link between sections Roseum and Sporotrichiella (through *F. arthrosporioides*). Nevertheless, in section Roseum, microconidia are typically absent, conidia are broad ellipsoid, characteristically of the same diameter as their length, comparatively narrow, and true chlamydospores are always absent. Surprisingly, *F. avenaceum* was not mentioned in Sherbakoff’s book. Conidia in *F. anguioides* are of diverse types, ranging from arthrosporial (short spindle shaped, with more or less rounded ends) to typically slightly curved or nearly straight and anguiform, 1- to 15-septate. No conspicuous sporodochia can be found. The color of conidia in the pseudopionnotal layer ranges from light pinkish cinnamon to cinnamon; arthrosporial conidia are common on aerial mycelium.

According to Booth [14], conidiophores of *F. avenaceum* have polyblastic conidiogenous cells, this being the reason for the location of this species in section Arthrosporiella together with *F. sporotrichioides*, *Fusarium semitectum,* and another *Fusarium* species, which produce conidia from polyblastic cells. *F. arthrosporioides* was placed in the section Gibbosum together with *Fusarium acuminatum* and *Fusarium equiseti*; these species are characterized as forming conidia in the aerial mycelium from simple phialides. However, *F. arthrosporioides* in contrast to other representatives of this section does not form chlamydospores.

Taxonomic views do not support the ability of *F. avenaceum* strains to produce polyphialides [16,18]. It would seem that the concept of the species has settled as member section Roseum. *F. avenaceum* do not produce microconidia and chlamydospores, and form unbranched and branched conidiophores with monophialides. The authors showed that macroconidia in *F. avenaceum* are generally scarce, but *F. arthrosporioides* produces oval-shaped, 0-3 septate, spindle-shaped macroconidia. However, since the production of microconidia in *F. avenaceum* is not well-defined, *F. arthrosporioides* was provisionally placed in *F. avenaceum* “until such time when it can be determined, if a clear-cut demarcation between the two exists” [16]. According to the book by Leslie and Summerell [17], *F. avenaceum* is able to produce fusoid 1-2-septate macroconidia and mesoconidia, and conidiogenous cells as monophialides and polyphialides. 

The investigations demonstrate that the systematic position of *F. avenaceum* is highly labile and subject to changes depending on which assumptions in the boundary of the diversity of morphological features were used [16,17,18]. Several *Fusarium* species, closely related to *F. avenaceum* morphologically, were gathered in *F. tricinctum* species complex according to molecular data [20,21,22]. 

In the phylogenetic analysis based on ribosomal intergenic spacer (IGS) and beta-tubulin DNA sequences, *F. avenaceum, F. arthrosporioides* and *F. anguioides* isolates form a species complex, which is closely related to *F. tricinctum* [10,23,24]. According to these results and chromatographic image analyses [10], *F. avenaceum* isolates can be divided into two main groups, of which main group II is more closely related to *F. arthrosporioides* than to other *F. avenaceum* isolates, while the two morphologically identified *F. anguioides* isolates did not form a phylogenetic group.

The main aim of the present work was to investigate, whether there are ISSR markers or beta-tubulin sequences, which are specific for morphologically identified *F. anguioides* isolates as compared to *F.avenaceum* and *F. arthrosporioides* isolates. Beta-tubulin sequences were chosen based on the previous promising results of Yli-Mattila et al. [23], which supported two main groups of *F. avenaceum* isolates. This is the first work, where molecular markers and DNA sequences specific for *F. anguioides* isolates have been found. In addition, the beta-tubulin sequences of *F. anguioides* isolates were compared to those of other closely related species found in GenBank, and more isolates of a new emerging *Fusarium* species were found in Asia. 

## 2. Results

### 2.1. Phenotypic Characters of Strains

All 26 *Fusarium* strains were moderately fast growing, and reached 65.3–85.2 mm in 7 days on potato sucrose agar (PSA) without any significant differences. The smallest colony diameter was detected in *F. anguioides* an39 strain (58.3 mm). Based on the sum of macro- and microscopic characteristics, all strains were assigned to three species: *F. avenaceum* (8 strains), *F. arthrosporioides* (4 strains), and *F. anguioides* (14 strains). 

**Figure 1:**
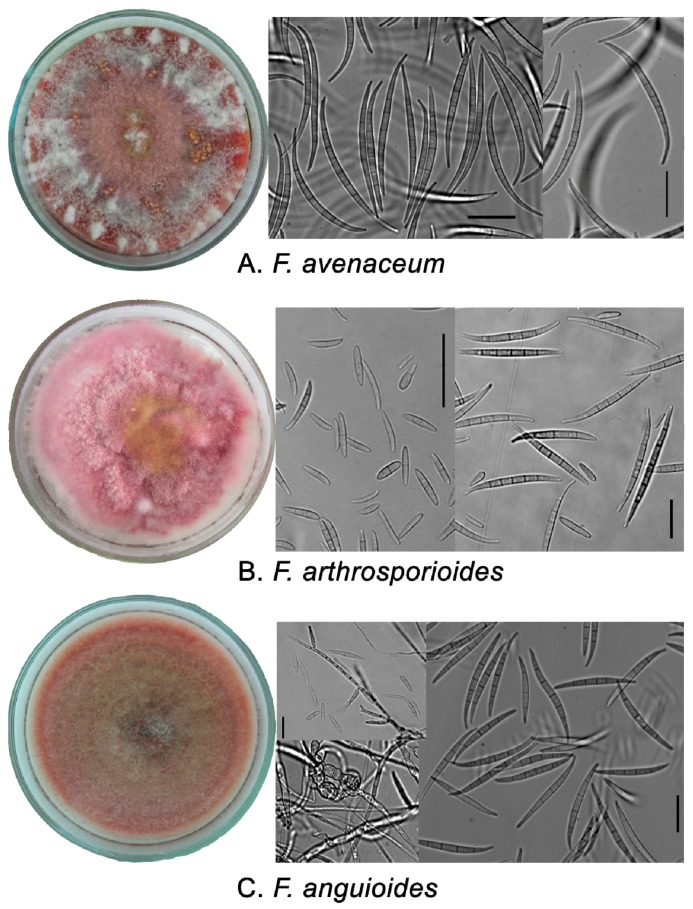
Typical morphological characters of *Fusarium avenaceum* strain MFG5864 (**A**), *Fusarium arthrosporioides* strain MFG116504 (**B**) and *Fusarium anguioides* strain MFG103100 (**C**) on PSA (14 days in the dark, 23 °С). The length of the scale bar is 20 μm.

The colonies of *F. avenaceum* were typically characterized with loosely fluffy to cottony aerial mycelia fringed with white and pink to carmine pigmentation (Figure 1). At the beginning, conidiophores were seen to arise laterally; secondary conidiophores had flexible rich branches. Conidiogenous cells are phialidic and slender. Bright orange sporodochia formed regularly in the aerial mycelium. Macroconidia from sporodochia were uniform with 5-7-septate, narrowly fusoid, curved with an elongated apical cell and a well-marked foot cell. The size of 5-septate macroconidia was 63.8 (51.1–80.0) × 4.1 (3.6–4.7) µm with an apical cell length of 17.1 (14.1–21.2) µm. In the aerial mycelium, fusoid 0-3-septate conidia (10–26 × 2.2–4.2 µm) occurred occasionally. Chlamydospores were not observed.

The colonies of *F. arthrosporioides* were fluffy to cottony with pink-rose aerial mycelia, and yellowish tinges, and pink pigmentation becoming red-brown with age. Conidiogenous cells were blastic and phialidic, often sympodial proliferating, sometimes with 2–3 conidiogenous loci. scattered sporulation started after a few days in aerial mycelium to give the surface of the colony a powdery appearance. Sometimes with age, pale brick sporodochia formed with 5-7-septate macroconidia. The size of 5-septate macroconidia was 56.2 (47.1–64.3) × 4.2 (3.8–4.7) µm with an apical cell length of 12.8 (10.4–16.9) µm. Typically, in the aerial mycelium and sporodochia, ovoid, fusoid, comma-like, 1-3-septate 8–30 × 3.2–5.0 µm sized conidia were formed. Chlamydospores were not observed.

The colonies of *F. anguioides* were loosely fluffy to cottony with carmine to ochre aerial mycelia, having red to brick tinges of pigmentation. Sporodochia in aerial mycelia were small, pale brick-colored, formed with 4-7-septate macroconidia. The size of 5-septate macroconidia was 54.9- (41.9–71.9) × 3.9 (3.0–5.0) µm with an apical cell length of 14.6 (10.4–21.2) µm. Conidiophores arose laterally (until 25 µm) and were whorled, thick-branched, and brick colored. Conidiogenous cells were blastic and phialidic, cylindrical, often sympodially proliferating, sometimes with 2–3 conidiogenous loci. scattered sporulation in aerial mycelia started after a few days to give the surface of the colony a powdery appearance. In the mycelia, ovoid, fusoid, comma-like, 1-3-septate, 11–38 × 3.2–5.0 µm sized conidia were formed. Intermediate chlamydospores and chlamydospore-like cells with a thick coat in the mycelium were observed in older strains.

### 2.2. Pathogenicity of Strains

**Figure 2:**
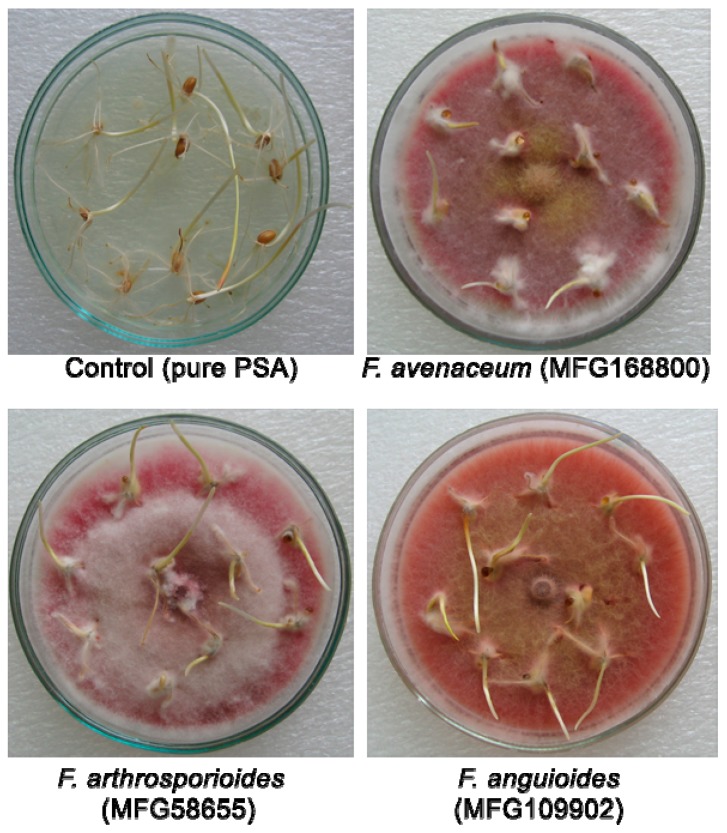
Pathogenicity of *Fusarium* strains toward winter wheat seedlings of cultivar Moskovskaya 39 (PSA, 10 days in the dark, 23 °С).

**Figure 3:**
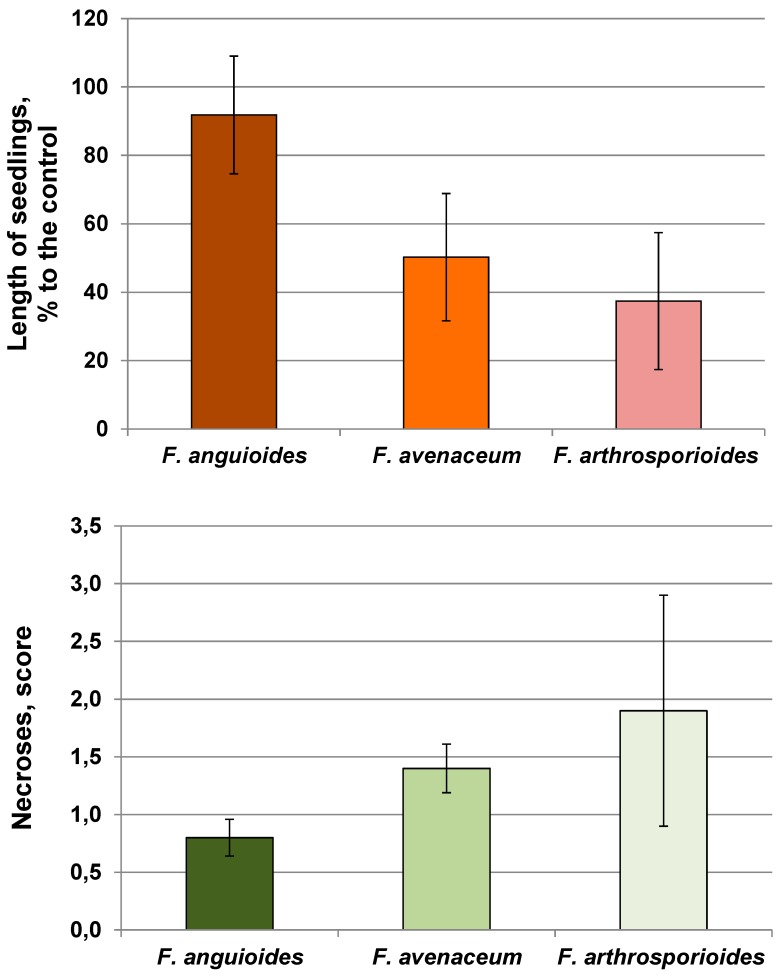
Effect of *Fusarium* spp. on the length and occurrence of necrosis of winter wheat seedlings of cultivar Moskovskaya 39.

The length of wheat seedlings on pure PSA (control) was on average 3.98 ± 0.23 cm without any symptoms of disease in the plant tissue. The negative effect of *F. avenaceum* and *F. arthrosporioides* strains on the length of seedlings and occurrence of necrosis was significantly higher as compared to that of *F. anguioides* strains (Figure 2 and Figure 3, Supplemental Table S1). A significant variation in the aggressiveness of *F. arthrosporioides* strains was found. The most pathogenic strain was *F. arthrosporioides* ar40, which demonstrated a high inhibition effect on the length of seedlings—7.6% as compared to control, and on average, score of necrosis of wheat tissue reached 2.6.

### 2.3. Molecular Diversity of Strains

Analyses of PCR results showed that all 21 strains produced clear positive reactions and an expected PCR product of 300 bp after amplification of their DNA with species-specific primer pair JIA f/r.

**Figure 4:**
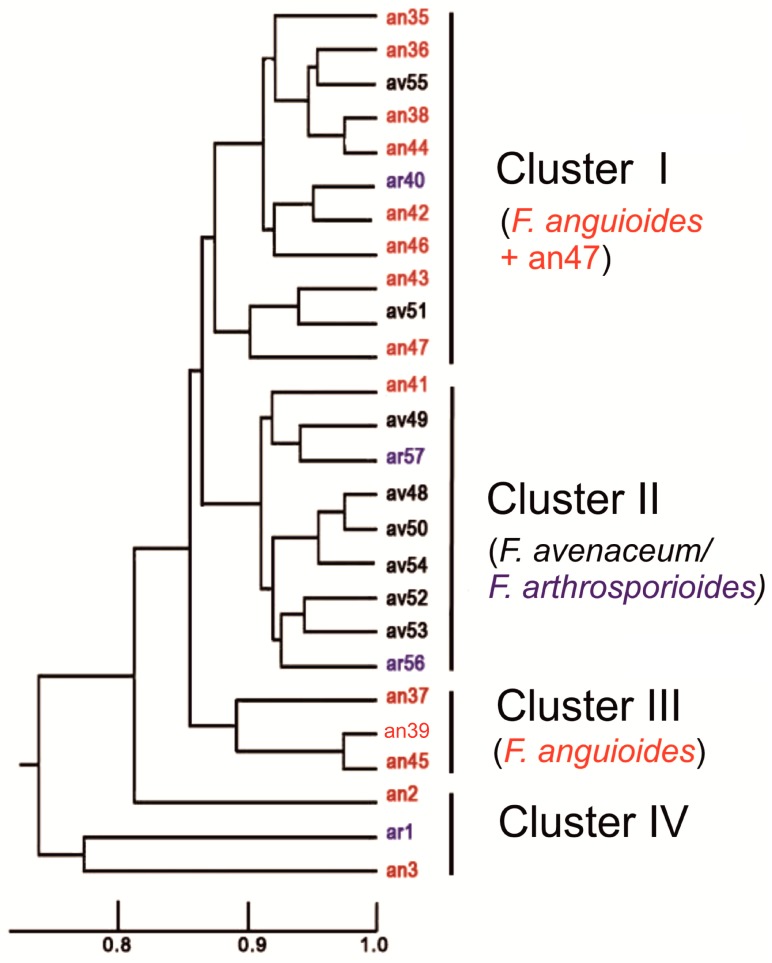
UPGMA dendrogram based on the results of ISSR fingerprinting patterns. The names of the strains were identified as different species according to their phenotypic characters marked by different colors (black—*F. avenaceum*, blue—*F. arthrosporioides,* and red—*F. anguioides*).

The results of DNA amplification with ISSR primers B, C, D, E, and G (Supplemental Figure S1) demonstrated the presence of polymorphic products ranging from six to nine informative bands. A UPGMA (unweighted pair group method with arithmetic mean) tree was constructed based on the sum of results obtained with all ISSR primers (Supplemental Table S2). In the UPGMA dendrogram (Figure 4), there were two big clusters (I and II) and two small clusters (III and IV). Most of the strains in cluster I comprised of *F. anguioides* (eight strains). Only one *F. avenaceum* strain (av51) and one *F. arthrosporioides* strain (ar40) originating from the Kirov region were present in this cluster. The second big cluster, cluster II, contained six *F. avenaceum* strains, two *F. arthrosporioides* strains (ar56 and ar57), and only one *F. anguioides* strain (an41). The third small cluster was represented by tree *F. anguioides* strains (an37, an39, and an45) from the north-western part of Russia, while another small cluster included one *F. anguioides* strain from Japan (an3), and one *F. arthrosporioides* strain (ar1). Another Japanese strain (an2) was not grouped to any cluster. We had problems in getting good ISSR amplification products with strains an2, ar1 and an3, which may explain, why they differed so clearly from other strains. The results of the UPGMA tree (Figure 4) were mainly supported with those of the PENNY consensus tree (Supplemental Figure S2), except for strain an36.

**Figure 5:**
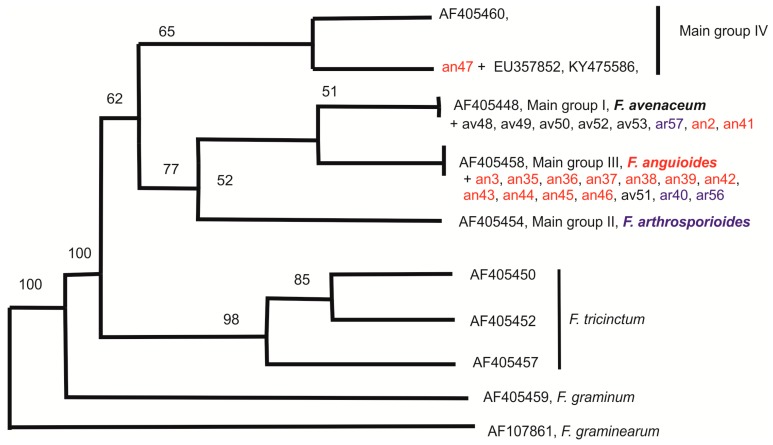
NJ consensus tree for beta-tubulin sequences of the strains. Only bootstrap values higher than 50% are shown. Additionally GenBank accessions no. of sequences from the article by Yli-Mattila et al. [23] are included.

**Figure 6:**
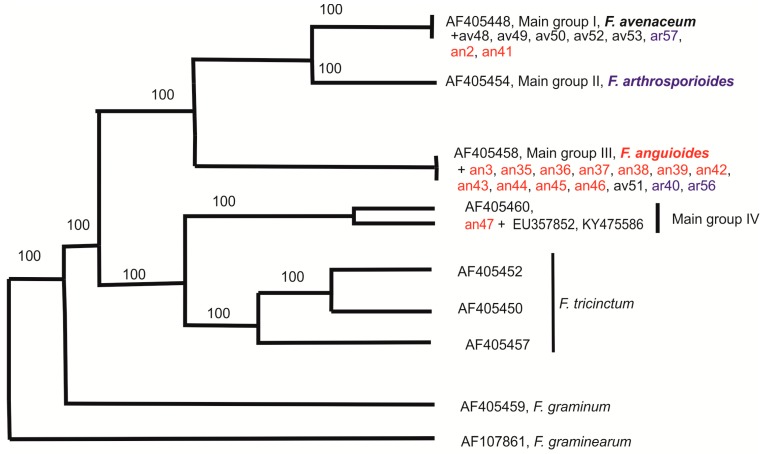
Parsimony consensus tree of 91 most parsimonious trees (221 steps) for beta-tubulin sequences of the strains. Only bootstrap values supported by more than 50% of the trees are shown. Additional GenBank accession numbers of sequences from the article by Yli-Mattila et al. [23] are included.

According to beta-tubulin DNA sequences, the strains can be divided into two groups (Figure 5). Five *F. avenaceum* strains (av48, av49, av50, av52, av53), one *F. arthrosporioides* strain (ar57), and two *F. anguioides* strains (an2, an41) had identical beta-tubulin sequences with accession number AF405448 of main group I [23]. Another big group was represented by eleven *F. anguioides* strains (an3, an35, an36, an37, an38, an39, an42, an43, an44, an45, an46), one *F. avenaceum* strain (av51), and two *F. arthrosporioides* strains (ar40, ar56), which had identical beta-tubulin sequences with accession number AF405458 of main group II [23]. Only one strain (an47), morphologically identified as *F. anguioides* was not grouped with other studied strains. In the neighbor joining (NJ) consensus tree (Figure 5) strain an47 together with identical beta-tubulin sequences of accession numbers EU357852 and KY475586 from Asia and similar beta-tubulin sequence AF405460 from Europe (main group IV) was closer to *F. avenaceum*, while in the parsimony consensus tree (Figure 6) this clade was closer to *F. tricinctum*.

## 3. Discussion

The morphological description of fungi closely related to *F. avenaceum* has been controversial [14,16,18]. *F. avenaceum*, *F. arthrosporioides*, and *F. anguioides* strains are characterized by high similarity, without distinct phenotypically separated boundaries. For these species, the formation of macroconidia with five septae and the range of variation in their size are highly similar. However, as can be seen from the description of species in the taxonomic systems by Gerlach and Nirenberg [15], for the characterization of related species of *Fusarium*, the size of the macroconidia does not have a significant diagnostic value. 

*F. arthrosporioides* occupies an intermediate position between these species and, indeed, can be referred to as belonging to both the Roseum and Artrosporiella sections [16]. *F. arthrosporioides* also produces bright orange sporodochia, but their formation is observed later than in *F. avenaceum*. *F. arthrosporioides* is able to produce microconidia on polyphialides with 2–3 conidiogenous loci; however, polyphialidic conidiogenous cells are detected rarely as compared to *F. anguioides*.

Sporodochia in *F. anguioides* are smaller, brick-red colored, and formed later than in *F. avenaceum*. *F. anguioides* on PSA and SNA media produces a more intense pigment of dark brown hue, compared to the other two species. 

For single-spore cultures of these *Fusarium* species, a relatively rapid degeneration after repeated sub-culturing is characteristic, which creates a problem for maintaining strains in the collection. Degenerated subcultures have white, sterile, tufted, aerial mycelia or merely a pionnotal layer. 

The pathogenicity of fungi toward plants is an important physiological characteristic. In our study, *F. avenaceum* and *F. arthrosporioides* strains were more aggressive, and they inhibited the length of wheat seedlings and caused greater necrosis than *F. anguioides*. One of the *F. anguioides* strains (an37) was nonpathogenic and it significantly stimulated seedling growth. A few years ago, *F. anguioides* was detected in Japan as a pathogen of *Sandersonia aurantiaca* bulbs [3]. Their morphological features are in accordance with our conception of this species. 

The results of the molecular-genetic study showed that most *F. anguioides* strains can be separated from most *F. avenaceum* strains based on ISSR results (big clusters I and II), while the three *F. arthrosporioides* strains did not gather to any cluster. Out of these two big clusters, three *F. anguioides* strains (an37, an39, an45) originated from north-western part of Russia to form their own cluster. The Japanese *F. anguioides* strains (an2, an3) and one *F. arthrosporioides* strain (ar1) were the most different from the rest of the analyzed strains, but it may be due to problems in getting ISSR products with some primers in these three strains. In the phylogenetic tree of beta-tubulin sequences, all analyzed strains form a species complex, which is clearly different from the *F. tricinctum* cluster. Analysis of beta-tubulin region sequences demonstrated the distribution of all analyzed strains, instead of an47, in two big groups. However, previous work by Yli-Mattila et al. [18] with species closely related to *F. avenaceum* has shown three existing main groups (I—*F. avenaceum*, II—*F. arthrosporioides*, and III—*F. anguioides*). 

Our beta-tubulin results supported the existence of groups I and III. Most of the morphologically typical *F. avenaceum* strains belong to main group I, while most of the morphologically typical *F. anguioides* strains belong to main group III. Main group III corresponds to clusters I and III in the UPGMA tree of ISSR patterns, while main group I corresponds to cluster II. We did not find any strains that would belong to main group II, which is connected to *F. arthrosporioides* [23]. In the present work, three *F. arthrosporioides* strains, ar40, ar56, and ar57, were spread in main groups I or III. There is only one different nucleotide in the partial beta-tubulin sequence between accession number, AF405448, of main group I containing mostly *F. avenaceum* strains and main group III containing mostly *F. anguioides* strains. 

*F. anguioides* strain an47 (rudbecia, Vladivostok, Russia) forms its own group differing from all other strains and has beta-tubulin sequence (submitted to GenBank) similarities with *F. avenaceum* strain accession no. AF405460 (*Ulmus scabra*, Austria). Interestingly, both of these strains, which were not isolated from cereals, were characterized by the high similarity of the beta-tubulin sequence. However, the clear correlation with host plant and geographic origin of the strains was not detected. More strains having identical beta-tubulin sequences with strain an47 were found by BLAST analysis, for example, accession no. KY475586 from China and EU357852 from Iran [25]. The host of KY475586 sequence was *Juglans regia* cv. Qingxiang (walnut), while the strain having the sequence EU357852 was isolated from Citrus [25]. Therefore, they are also from dicotyledonous plants.

Species populations occurring in different habitats can exhibit physiological, morphological, and genetic differences in response to contrasting environmental conditions. Kulik et al. [26] and Stakheev et al. [27] carried out phylogenetic analyses based on five and four individual genes, respectively, and combined data sets, that showed lack of clear phylogenetic structure within *F. avenaceum* in relation to the host and geographic origin. The results obtained by Gräfenhan et al. [6] revealed that the strains isolated from warmer areas of Asia represented an individual lineage that together formed a closely related sister clade to *F. avenaceum*. The future analyses of more strains from the Asian region may lead to describing of new phylogenetically related groups within the *F. avenaceum* and *F. anguioides* species. 

Two strains of *F. anguioides,* an38 and an44, from one grain sample of barley, and the *F. anguioides* strain an41 from another grain sample of barley harvested from neighboring fields in the Kaliningrad region were taken for comparison of their genetic diversity. According to ISSR fingerprinting patterns and beta-tubulin sequences, the strains, an38 and an44, from the Kaliningrad region have high similarity, being part of cluster I in the UPGMA tree and the main group I in the Neighbor Joining (NJ) tree, but they are not clones. *F. anguioides* strain an41 is located in cluster II, and main group III. 

Two strains, *F. avenaceum* av49 and *F. anguioides* ang46, caught from air in Poland, were also included in this study for comparison with the strains isolated from plants. In accordance to the morphological identification, the strains from Poland have distributed in the clusters of UPGMA dendrogram and groups of the NJ tree for beta-tubulin sequences representing mostly *F. avenaceum* or *F. anguioides* strains.

Based on our results *F. anguioides* may be a real species. The morphological identification of most strains was supported by molecular data, but our present results of ISSR and the beta-tubulin sequences analyses confirmed that only one of the two Japanese *F. anguioides* strains obtained from the BBA collection was *F. anguioides*. The separation between these strains is also supported by previous dot blot hybridization data of Yli-Mattila et al. [23].

The identification of the *F. arthrosporioides* strains in the present work according to morphological features was not clearly supported by our results of the phylogenetic analysis. According to the pathogenicity to wheat seedlings, these strains were identical to *F. avenaceum* strains. The formation of microconidia, polyphialides, and chlamydospores have been chosen as basic characteristics for differentiation of *F. arthrosporioides* strains from macromorphologically similar *F. avenaceum* strains. The presence of these described characteristics is not enough for separation of these closely related species, because polyphialides and different kinds of conidia have been observed in some strains of *F. avenaceum*, as it was postulated in the book by Leslie and Summerell [17]. Recently, it has been noticed by Gräfenhan et al. [6] that several reference *F. arthrosporioides* strains from colder climates formed a distinct lineage in the main *F. avenaceum* clade with moderate bootstrap support (72%) in the phylogenetical analysis inferred from partial DNA sequences of the *ac11* and *tef-1α* genes. In addition, Nalim et al. [25] found bootstrap support (99%) on the basis of sequences of *tef-1α* and beta-tubulin for a small clade containing two strains from Finland and Egypt, which was named as *F. avenaceum-arthrosporioides.* However, the beta-tubulin sequences of these strains were different from the beta-tubulin sequences of main group II [23] containing *F. arthrosporioides* strains.

The taxonomic status of *F. avenaceum* and closely related species should be confirmed by genome sequences of strains originating from diverse regions and hosts. The published whole-genome sequence of *F. avenaceum* [28] is a useful basis for understanding the next steps of clear taxonomical status of closely related fungi. Future research will contribute to further insights into pathogenicity, phylogeny, and adaptation of *F. avenaceum* and closely related species in diverse environments.

## 4. Materials and Methods 

### 4.1. Strains of Fusarium

The list of *F. avenaceum*, *F. arthrosporioides,* and *F. anguioides* strains are shown in Table 1. A total of 21 strains were isolated by the authors from plants, which were collected from different regions of Russia. Kaliningrad, Vologda, Novgorod, Kirov, Pskov, and Leningrad regions belonging to the north-western part of Russia, whereas Moscow, Krasnodar, and Tyumen regions are located in the central, south European, and west Siberian parts of Russia, respectively. Two strains (MFG58656 and MFG58657) were received from Paweł Serbiak from Institute of Plant Genetics, Poznań, Poland. Three strains were obtained from the collection of the Institute of Microbiology, Berlin, Germany. All *Fusarium* strains used in the study were single-spored. The strains are maintained in the collection of the Laboratory of Mycology and Phytopathology (All-Russian Institute of Plant Protection, St. Petersburg, Russia).

### 4.2. Morphological Analyses

Phenotypic characterization of the 26 strains was performed macro- and microscopically when the fungal cultures were cultivated on synthetic nutrient poor agar (SNA; Nirenberg [29]) and potato sucrose agar (PSA) at 23–25 °C under dark conditions. Morphological observations included the type of growth of the mycelium, pigmentation presence, and characteristics of sporodochia, as also the shape, degree of septation, size of macro- and microconidia, conidiophore length and branching patterns, and presence or absence of chlamydospores [18,19,30]. Mycelial growth rate was calculated as mean value of diameters of the colony measured in two perpendicular directions around the colony grown on PSA. All measurements were made in duplicate. Micromorphological features were examined and observed using an AxioVision Viewer 4.8 microscope (Carl Zeiss). The length and width of at least 30–50 conidia were measured, and the mean and range values were calculated. 

### 4.3. The Analyses of Pathogenicity

Pathogenicity of 15 strains (Supplemental Table S1) was determined by the modified laboratory method [31]. Grains of winter wheat Moskovskaya 39 were surface sterilized with 70% ethanol and soaked for 24 h in sterile water. The grains with swollen germs were then placed over the fungal culture grown for a week on PSA (10 grains per Petri dish) in triplicate. In the control, grains were placed on the PSA without fungi. After a week of incubation in the dark at 23–25 °C, the length of seedlings was measured, and their state scored using a four-grade scale: 0—healthy seedling; 1—dotted tissue necrosis; 2—necrosis of about 50% of the tissue area; and 3—complete death. In every variant, the length of each seedling was determined and the average value for each experimental variant was calculated. The decrease in seedling length caused by the effect of fungi was assayed as a percentage of the average length in the control. The experiments were repeated in duplicate.

### 4.4. DNA Extraction, PCR and Sequencing

DNA was extracted from the 26 *Fusarium* strains by using the CTAB (cetyl trimethylammonium bromide) method [32] with some variations. All DNA samples were stored at −20 °C prior to PCR. The DNA quality of each strain was confirmed by using ITS1/ITS4 primers [33]. The species-specific primer pair JIA f/r [34] (Supplemental Table S3), which is specific for *F. avenaceum*, was used in PCR for confirmation of the morphological identification of strains.

ISSR analyses with ISSR primers B, C, D, E, and G (Supplemental Table S3) were performed according to protocols and conditions of reactions as described by Stenglein and Balatti [35]. Amplification of DNA with primers was performed in a PTC-200 DNA Engine thermal cycler (MJ Research). Aliquots (10 μL) of each PCR product were analyzed by electrophoresis in a TBE buffer in 1.5% agarose gel. The present and anticipated size of the PCR products of 26 strains were visualized by using the Fluorchem^TM^ Advanced Fluorescence, Chemiluminescence and Visible Light Imaging (Alpha Innotech Corporation).

The partial beta-tubulin region of 23 strains, including all 14 *F. anguioides* strains, was amplified and sequenced as described by Yli-Mattila et al. [23] (Supplemental Table S3). The partial beta-tubulin sequences of the strains were also compared to those of known *Fusarium* sequences found in GenBank. *F. graminearum* strain gr1 (accession no. AF107861) was used as an outgroup. UPGMA (unweighted pair group method with arithmetic mean) and Neighbor Joining (NJ) consensus trees were prepared by using the PHYLIP (the PHYLogeny Inference Package) program as described by Yli-Mattila et al. [23]. The programs SEQBOOT (random seed 81, total number of replicates 100), DNADIST (Kimura), DNAPARS, PENNY and CONSENSE of PHYLIP were also used.

## 5. Conclusions

High genetic diversity was revealed in *F. avenaceum* and related species. *F. avenaceum* and *F. anguioides* were characterized as separate species and most strains of these species could be identified by morphological and molecular data. The four *F. arthrosporioides* strains did not form their own group in molecular analyses, although in previous investigations *F. arthrosporioides*-specific molecular markers have been found. Instead, molecular markers and DNA sequences specific for *F. anguioides* isolates were found. The obtained results also suggest the existence of a new emerging *Fusarium* species distributed in Asia. A larger systematic and phylogenetic study is necessary to clarify the taxonomic status of the *F. avenaceum* clade using more strains, reliable accession number information and whole-genome sequences. This is likely to contribute to further insights into their pathogenicity, phylogeny and adaptation to diverse environments within *F. tricinctum* species complex.

## Figures and Tables

**Table 1:** Fusarium strains used in the study and identified based on morphological characters.

No.	Catalogue no.*	*Fusarium* sp.	Origin	Substrate	Year	Strain no.
1	MFG103100	*F. anguioides*	Kaliningrad, Russia	oat, grain	2007	an35
2	MFG109902	*F. anguioides*	Vologda, Russia	wheat, grain	2008	an36
3	MFG112804	*F. anguioides*	Novgorod, Russia	barley, grain	2008	an37
4	MFG115014	*F. anguioides*	Kaliningrad, Russia	barley, grain	2008	an38
5	MFG114003	*F. anguioides*	Novgorod, Russia	oat, grain	2008	an39
6	MFG116504	*F. arthrosporioides*	Kirov, Russia	barley, grain	2008	ar40
7	MFG114605	*F. anguioides*	Kaliningrad, Russia	barley, grain	2008	an41
8	MFG119913	*F. anguioides*	Kirov, Russia	oat, grain	2008	an42
9	MFG118902	*F. anguioides*	Kirov, Russia	oat, grain	2008	an43
10	MFG115015	*F. anguioides*	Kaliningrad, Russia	barley, grain	2008	an44
11	MFG108904	*F. anguioides*	Pskov, Russia	barley, grain	2008	an45
12	MFG58657	*F. anguioides*	Poland	air	2013	an46
13	MFG58314	*F. anguioides*	Vladivostok, Russia	rudbeckia, leaves	2010	an47
14	MFG118702	*F. avenaceum*	Pskov, Russia	barley, grain	2009	av48
15	MFG58656	*F. avenaceum*	Poland	air	2013	av49
16	MFG151200	*F. avenaceum*	Leningrad, Russia	oat, grain	2011	av50
17	MFG58640	*F. avenaceum*	Moscow, Russia	wheat, grain	2004	av51
18	MFG168800	*F. avenaceum*	Leningrad, Russia	oat, grain	2011	av52
19	MFG118401	*F. avenaceum*	Pskov, Russia	oat, grain	2009	av53
20	MFG58426	*F. avenaceum*	Krasnodar, Russia	barley, grain	2011	av54
21	MFG117309	*F. avenaceum*	Kirov, Russia	oat, grain	2008	av55
22	MFG58654	*F. arthrosporioides*	Tyumen, Russia	barley, grain	2013	ar56
23	MFG58655	*F. arthrosporioides*	Leningrad, Russia	oat, grain	2013	ar57
24	BBA64215	*F. arthrosporioides*	unknown	unknown	un-known	ar1
25	BBA63598	*F. anguioides*	Japan	pea	1928	an2
26	BBA69055	*F. anguioides*	Japan	wheat, grain	1994	an3

* MFG—the numbers of strains in the collection of the Laboratory of Mycology and Phytopathology (All-Russian Institute of Plant Protection, St. Petersburg, Russia); BBA—the numbers of strains in the collection of Federal Biological Research Center for Agriculture and Forestry (Institute of Microbiology, Berlin, Germany).

## Supplementary Materials

The following are available online at http://www.mdpi.com/2076-0817/7/4/94/s1, Figure S1: ISSR photos, Figure S2: PENNY consensus tree of ISSR data, Table S1: The analyses of pathogenicity, Table S2: ISSR data matrix, Table S3: Primers used in the present work.
