# Supplementary material for: Morphological and Molecular Variation Between Fusarium avenaceum, Fusarium arthrosporioides and Fusarium anguioides Strains
Source: Pathogens. 2018 Nov 29;7(4):94. doi: 10.3390/pathogens7040094 (PMC6313738; doi:10.3390/pathogens7040094)

Table S1. **The analyses of pathogenicity of strains**

**Catalogue no. of strain**	**Seedling length, mm**	**Necroses, score**	***Fusarium sp.***
MFG103100	83,3	0,6	*F. anguioides*
MFG109902	69,4	1,2	*F. anguioides*
MFG112804	291,9	0,1	*F. anguioides*
MFG115014	45,2	1,4	*F. anguioides*
MFG114003	65,3	0,2	*F. anguioides*
MFG119913	69,8	0,7	*F. anguioides*
MFG118902	67,8	0,6	*F. anguioides*
MFG115015	45,2	1,4	*F. anguioides*
MFG108904	87,8	0,2	*F. anguioides*
**The average±SD**[a]	**91,7±24,0**	**0,7±0,16**	
MFG118702	76,2	1	*F. avenaceum*
MFG58640	41,5	1,3	*F. avenaceum*
MFG168800	33,1	2,1	*F. avenaceum*
**The average±SD**	**50,3±10,8**	**1,5±0,27**	
MFG58654	28,9	1,8	*F. arthrosporioides*
BBA64215	57,2	1,2	*F. arthrosporioides,*
MFG116504	26	2,6	*F. arthrosporioides*
**The average±SD**	**37,4±0,9**	**1,8±0,3**	

[a]SD – standard deviation

Table S2. **ISSR data matrix:**

```
26  39
an35        111001001100101001000001101001110101110
an36        111001001100101011010001001001001101111
an37        100111001100111111000010001001110101110
an38        110001001100101011000001101001101101111
an39        000111001110100101000011001001101101111
ar40        110001001100101011100111101011101111111
an41        110001001100110010001000001100101101111
an42        100101001100101011000111101011110111111
an43        111001011110101001000111001011001101111
an44        110001001100101011000001101001100111111
an45        000111001100100101000011001001001101111
an46        100001101100101001100011101001101101111
an47        100001011100110001001110001010001111111
av48        111001001100111101001010001100000101111
av49        100001001100111011001010001100101111110
av50        111001001100111001001010001100001101111
av51        101001011110100001110111001011101111111
av52        111001001100101101011001001100101101111
av53        110001001100101101011010011000101101111
av54        111001101101111001001010001100101101111
av55        110001001110101011010001001001101111111
av56        100001001101111001011001001100101101111
av57        100001001100110101001010011100101111111
ar1         00000110??????000100100000100100110001 1
an2         100001001101100101000000011100001100010
an3         000001100100001001001000101000010110000
```

Table S3. **Primers used in the present work**

ISSR primers:

B (ag)8 tg

C (ag)8 cg

D (ag)8

E ctc (gt)8

F ct (ga)8

G gag (caa)5

JIA primers (300 bp product, specific for *F. avenaceum/F. arthrosporioides*):

JIAf GCTAATTCTTAACTTACTAGGGGCC

JIAr CTGTAATAGGTTATTTACATGGGCG

Primers for amplification and sequencing partial beta-tubulin sequences:

T1 AACATGCGTGAGATTGTAAGT

T22 TCTGGATGTTGTTGGGAATC

tub-conrev T22 TGACCGAAAACGAAGTTGTC

Figure S1. **Photos of gels** with products of amplification of DNA with ISSR primers

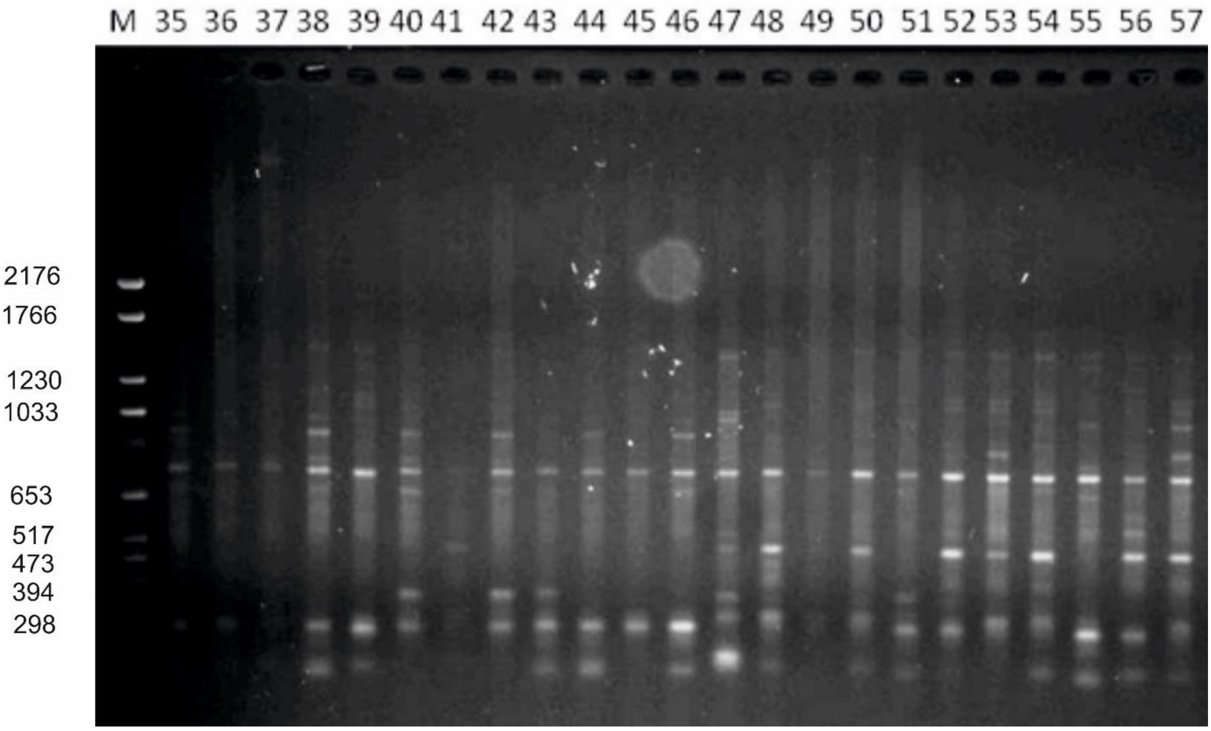

ISSR products with primer E in an, av and ar strains 35-57 as compared to molecular weight standard (M)

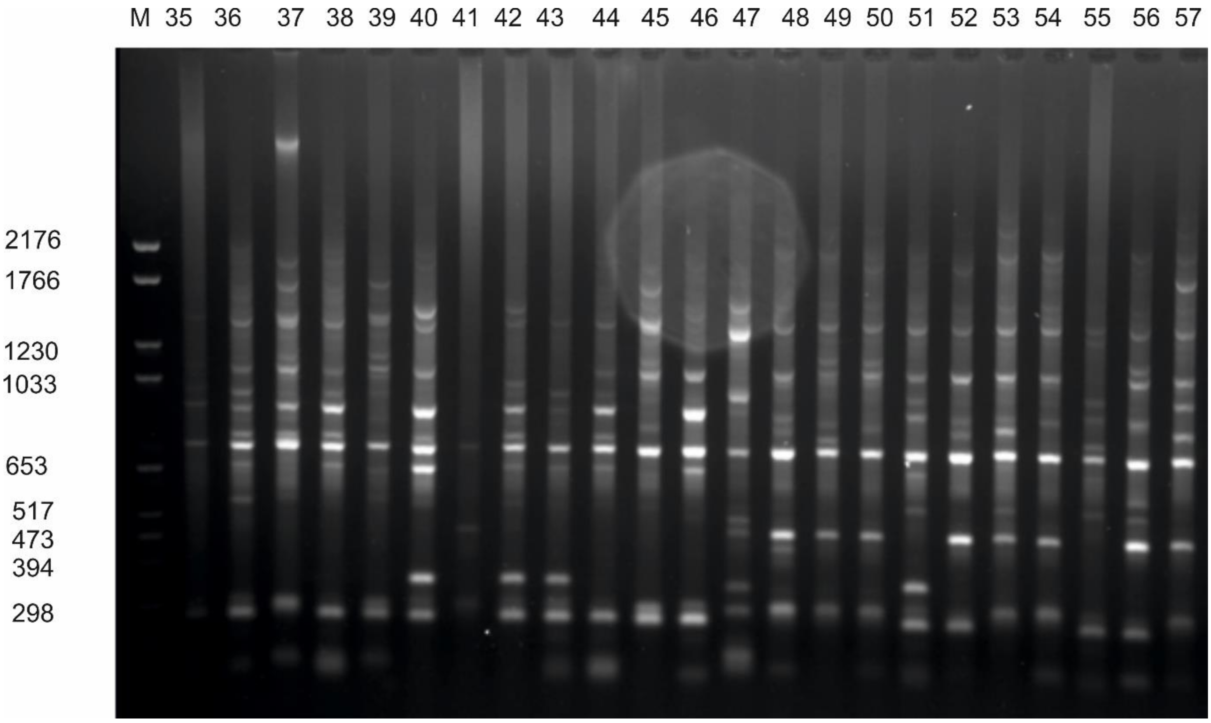

ISSR products with primer D in an, av and ar strains 35-57 as compared to molecular weight standard (M)

Figure S2. **PENNY consensus tree of ISSR data**. 24 most parsimonious trees with 112 steps were found.

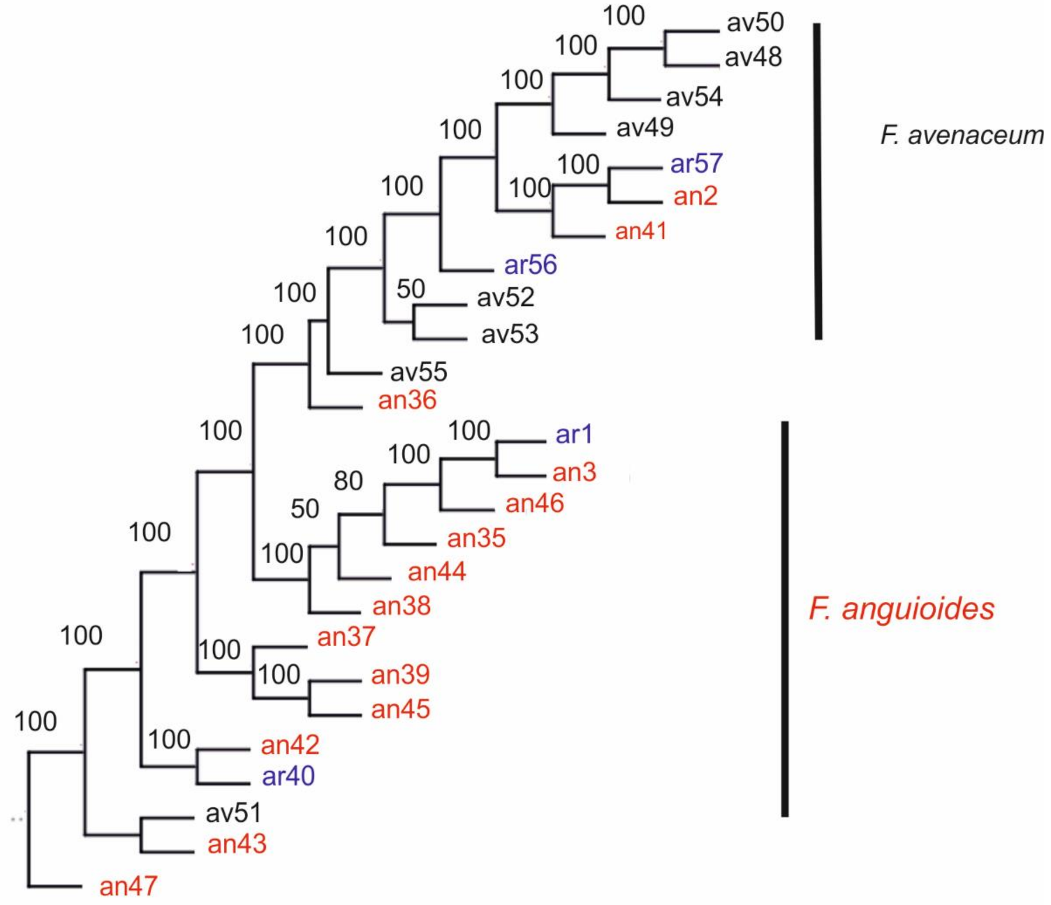

Supplement: Supplementary file 1 [file pathogens-07-00094-s001.pdf]
